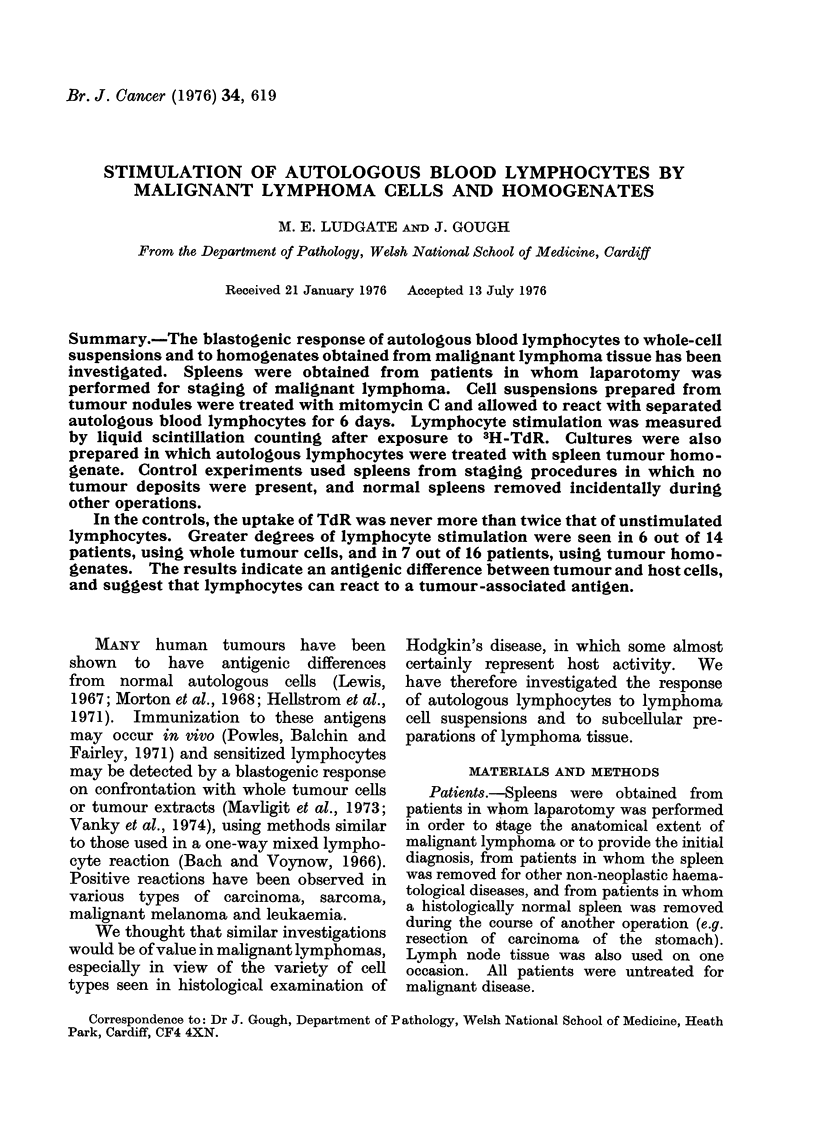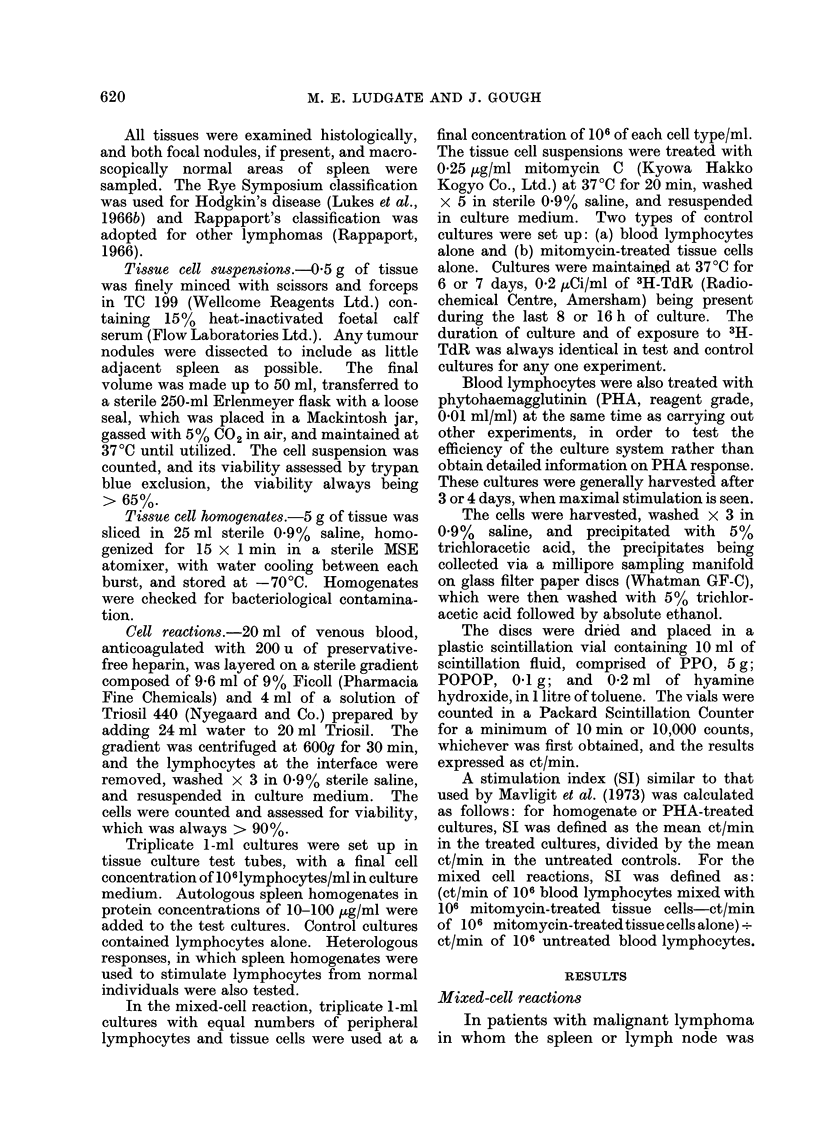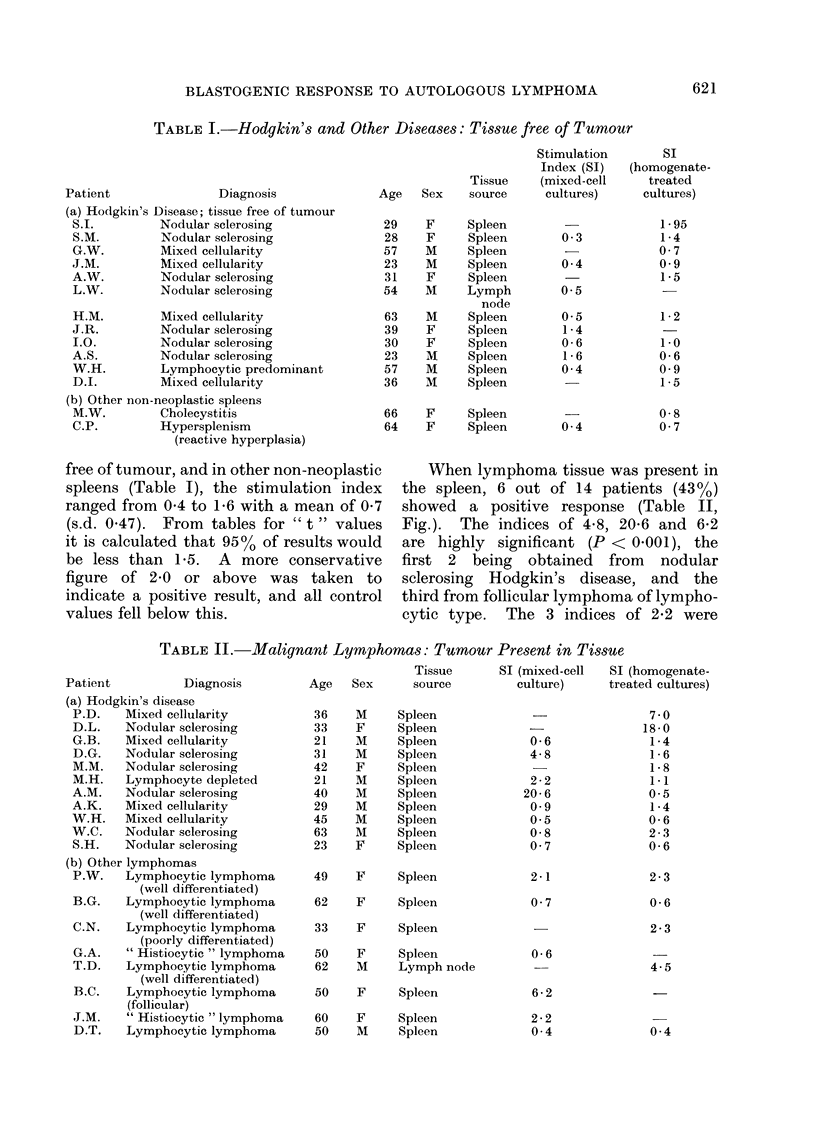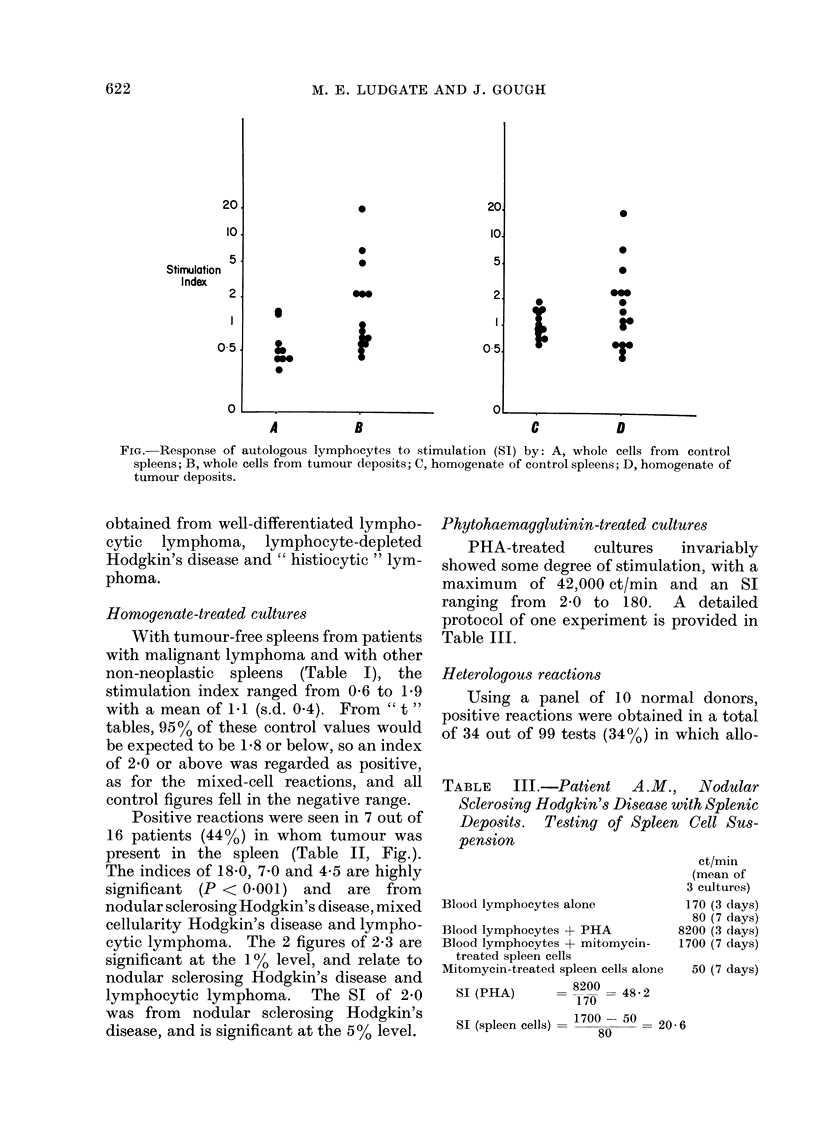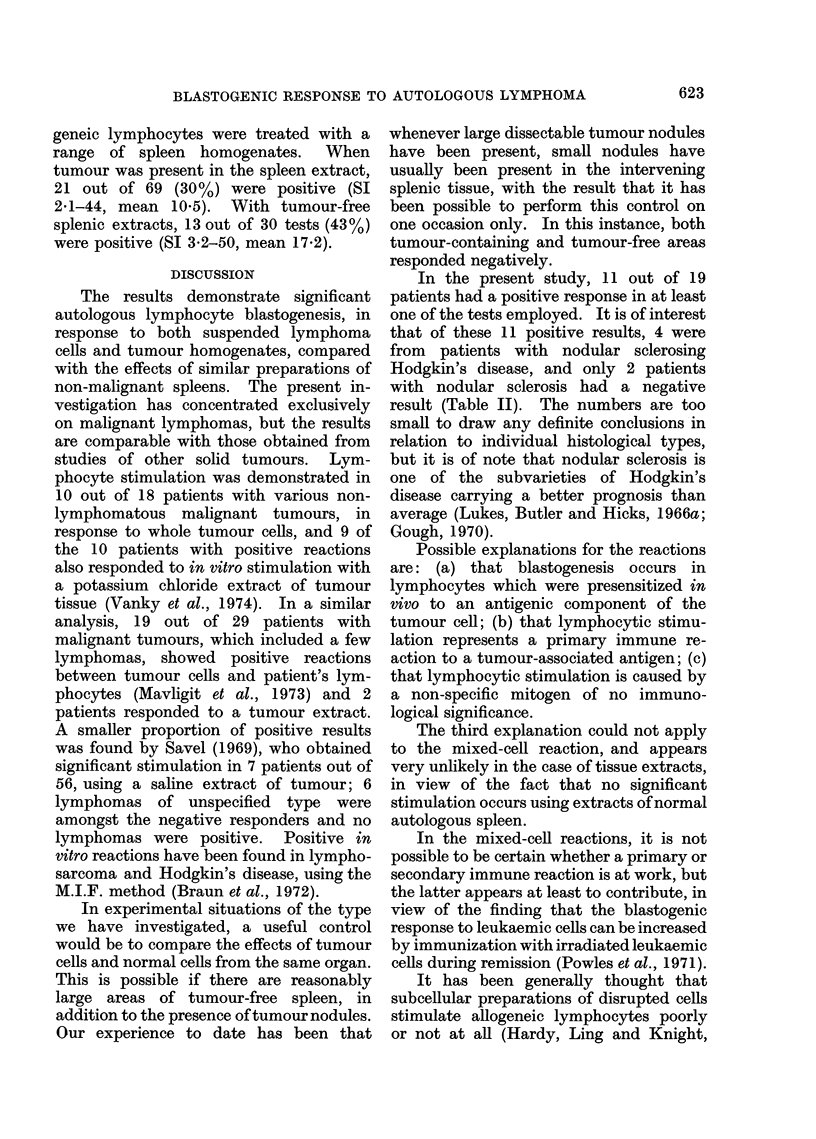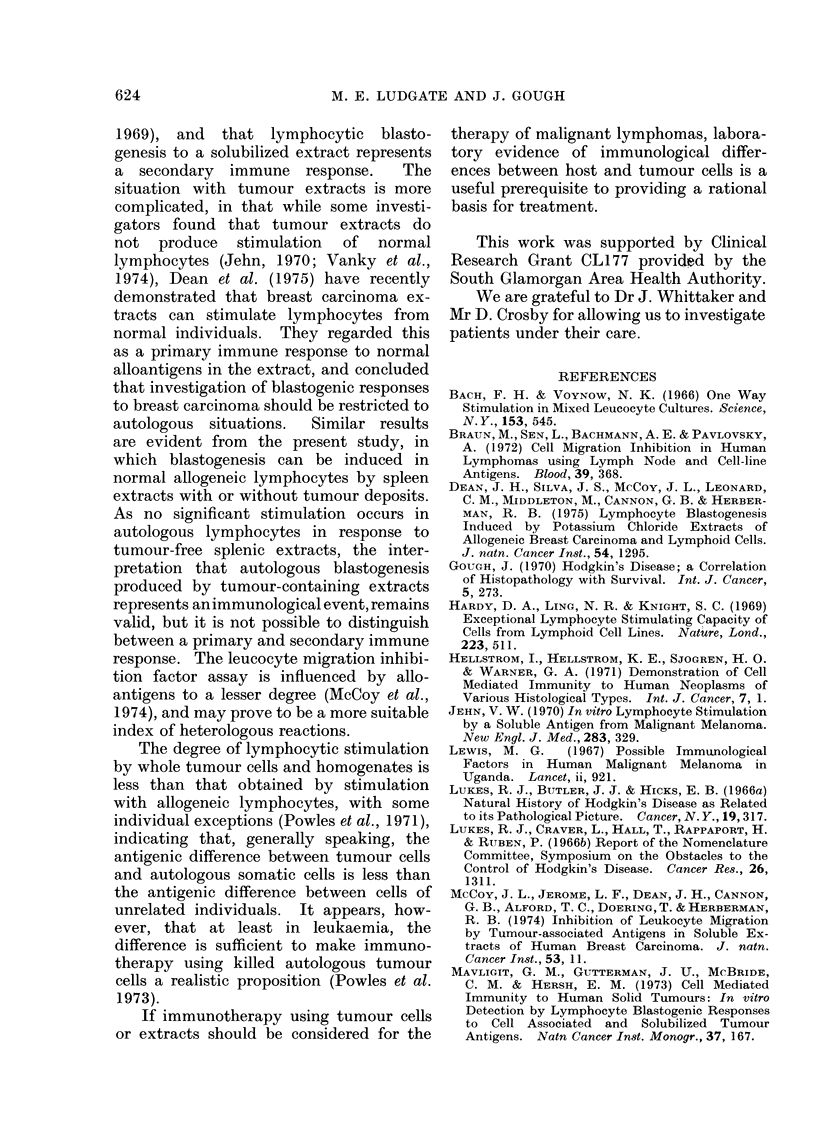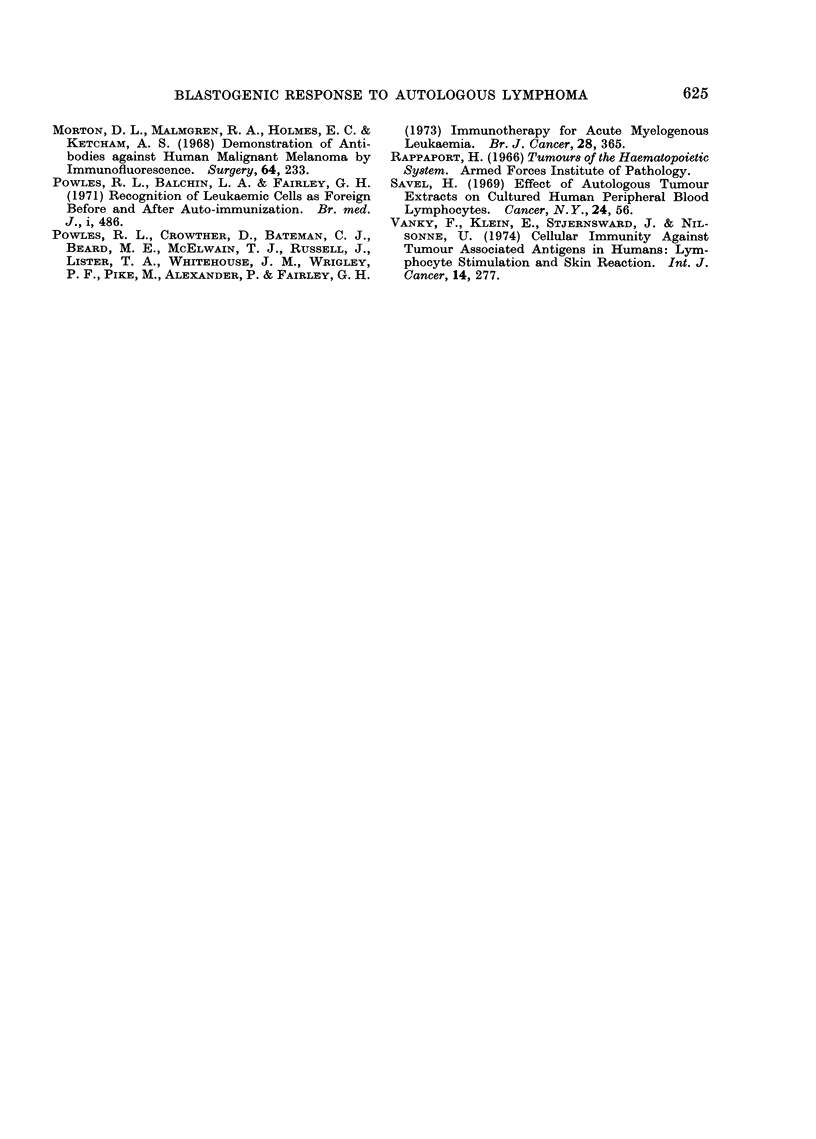# Stimulation of autologous blood lymphocytes by malignant lymphoma cells and homogenates.

**DOI:** 10.1038/bjc.1976.222

**Published:** 1976-12

**Authors:** M. E. Ludgate, J. Gough

## Abstract

The blastogenic response to autologous blood lymphocytes to whole-cell suspensions and to homogenates obtained from malignant lymphoma tissue has been investigated. Spleens were obtained from patients in whom laparotomy was performed for staging of malignant lymphoma. Cell suspensions prepared from tumour nodules were treated with mitomycin C and allowed to react with separated autologous blood lymphocytes for 6 days. Lymphocyte stimulation was measured by liquid scintillation counting after exposure to 3H-TdR. Cultures were also prepared in which autologous lymphocytes were treated with spleen tumour homogenate. Control experiments used spleens from staging procedures in which no tumour deposits were present, and normal spleens removed incidentally during other operations. In the controls, the uptake of TdR was never more than twice that of unstimulated lymphocytes. Greater degrees of lymphocyte stimulation were seen in 6 out of 14 patients, using whole tumour cells, and in 7 out of 16 patients, using tumour homogenates. The results indicate an antigenic difference between tumour and host cells, and suggest that lymphocytes can react to a tumour-associated antigen.


*Br. J. Cancer* (1976) **34**, 619

### STIMULATION OF AUTOLOGOUS BLOOD LYMPHOCYTES BY MALIGNANT LYMPHOMA CELLS AND HOMOGENATES

M. E. LUDGATE AND J. GOUGH

*From the Department of Pathology, Welsh National School of Medicine, Cardiff*

Received 21 January 1976 Accepted 13 July 1976

**Summary.—The blastogenic response of autologous blood lymphocytes to whole-cell suspensions and to homogenates obtained from malignant lymphoma tissue has been investigated. Spleens were obtained from patients in whom laparotomy was performed for staging of malignant lymphoma. Cell suspensions prepared from tumour nodules were treated with mitomycin C and allowed to react with separated autologous blood lymphocytes for 6 days. Lymphocyte stimulation was measured by liquid scintillation counting after exposure to $^3$H-TdR. Cultures were also prepared in which autologous lymphocytes were treated with spleen tumour homogenate. Control experiments used spleens from staging procedures in which no tumour deposits were present, and normal spleens removed incidentally during other operations.**

**In the controls, the uptake of TdR was never more than twice that of unstimulated lymphocytes. Greater degrees of lymphocyte stimulation were seen in 6 out of 14 patients, using whole tumour cells, and in 7 out of 16 patients, using tumour homogenates. The results indicate an antigenic difference between tumour and host cells, and suggest that lymphocytes can react to a tumour-associated antigen.**

MANY human tumours have been shown to have antigenic differences from normal autologous cells (Lewis, 1967; Morton *et al.*, 1968; Hellstrom *et al.*, 1971). Immunization to these antigens may occur *in vivo* (Powles, Balchin and Fairley, 1971) and sensitized lymphocytes may be detected by a blastogenic response on confrontation with whole tumour cells or tumour extracts (Mavligit *et al.*, 1973; Vanky *et al.*, 1974), using methods similar to those used in a one-way mixed lymphocyte reaction (Bach and Voynow, 1966). Positive reactions have been observed in various types of carcinoma, sarcoma, malignant melanoma and leukaemia.

We thought that similar investigations would be of value in malignant lymphomas, especially in view of the variety of cell types seen in histological examination of Hodgkin's disease, in which some almost certainly represent host activity. We have therefore investigated the response of autologous lymphocytes to lymphoma cell suspensions and to subcellular preparations of lymphoma tissue.

### MATERIALS AND METHODS

*Patients.*—Spleens were obtained from patients in whom laparotomy was performed in order to stage the anatomical extent of malignant lymphoma or to provide the initial diagnosis, from patients in whom the spleen was removed for other non-neoplastic haematological diseases, and from patients in whom a histologically normal spleen was removed during the course of another operation (*e.g.* resection of carcinoma of the stomach). Lymph node tissue was also used on one occasion. All patients were untreated for malignant disease.

Correspondence to: Dr J. Gough, Department of Pathology, Welsh National School of Medicine, Heath Park, Cardiff, CF4 4XN.

All tissues were examined histologically, and both focal nodules, if present, and macroscopically normal areas of spleen were sampled. The Rye Symposium classification was used for Hodgkin's disease (Lukes *et al.*, 1966*b*) and Rappaport's classification was adopted for other lymphomas (Rappaport, 1966).

*Tissue cell suspensions.*—0·5 g of tissue was finely minced with scissors and forceps in TC 199 (Wellcome Reagents Ltd.) containing 15% heat-inactivated foetal calf serum (Flow Laboratories Ltd.). Any tumour nodules were dissected to include as little adjacent spleen as possible. The final volume was made up to 50 ml, transferred to a sterile 250-ml Erlenmeyer flask with a loose seal, which was placed in a Mackintosh jar, gassed with 5% $CO_2$ in air, and maintained at 37°C until utilized. The cell suspension was counted, and its viability assessed by trypan blue exclusion, the viability always being $> 65\%$.

*Tissue cell homogenates.*—5 g of tissue was sliced in 25 ml sterile 0·9% saline, homogenized for 15 × 1 min in a sterile MSE atomixer, with water cooling between each burst, and stored at −70°C. Homogenates were checked for bacteriological contamination.

*Cell reactions.*—20 ml of venous blood, anticoagulated with 200 u of preservative-free heparin, was layered on a sterile gradient composed of 9·6 ml of 9% Ficoll (Pharmacia Fine Chemicals) and 4 ml of a solution of Triosil 440 (Nyegaard and Co.) prepared by adding 24 ml water to 20 ml Triosil. The gradient was centrifuged at 600*g* for 30 min, and the lymphocytes at the interface were removed, washed × 3 in 0·9% sterile saline, and resuspended in culture medium. The cells were counted and assessed for viability, which was always $> 90\%$.

Triplicate 1-ml cultures were set up in tissue culture test tubes, with a final cell concentration of $10^6$ lymphocytes/ml in culture medium. Autologous spleen homogenates in protein concentrations of 10–100 μg/ml were added to the test cultures. Control cultures contained lymphocytes alone. Heterologous responses, in which spleen homogenates were used to stimulate lymphocytes from normal individuals were also tested.

In the mixed-cell reaction, triplicate 1-ml cultures with equal numbers of peripheral lymphocytes and tissue cells were used at a final concentration of $10^6$ of each cell type/ml. The tissue cell suspensions were treated with 0·25 μg/ml mitomycin C (Kyowa Hakko Kogyo Co., Ltd.) at 37°C for 20 min, washed × 5 in sterile 0·9% saline, and resuspended in culture medium. Two types of control cultures were set up: (a) blood lymphocytes alone and (b) mitomycin-treated tissue cells alone. Cultures were maintained at 37°C for 6 or 7 days, 0·2 μCi/ml of $^3$H-TdR (Radiochemical Centre, Amersham) being present during the last 8 or 16 h of culture. The duration of culture and of exposure to $^3$H-TdR was always identical in test and control cultures for any one experiment.

Blood lymphocytes were also treated with phytohaemagglutinin (PHA, reagent grade, 0·01 ml/ml) at the same time as carrying out other experiments, in order to test the efficiency of the culture system rather than obtain detailed information on PHA response. These cultures were generally harvested after 3 or 4 days, when maximal stimulation is seen.

The cells were harvested, washed × 3 in 0·9% saline, and precipitated with 5% trichloracetic acid, the precipitates being collected via a millipore sampling manifold on glass filter paper discs (Whatman GF-C), which were then washed with 5% trichloracetic acid followed by absolute ethanol.

The discs were dried and placed in a plastic scintillation vial containing 10 ml of scintillation fluid, comprised of PPO, 5 g; POPOP, 0·1 g; and 0·2 ml of hyamine hydroxide, in 1 litre of toluene. The vials were counted in a Packard Scintillation Counter for a minimum of 10 min or 10,000 counts, whichever was first obtained, and the results expressed as ct/min.

A stimulation index (SI) similar to that used by Mavligit *et al.* (1973) was calculated as follows: for homogenate or PHA-treated cultures, SI was defined as the mean ct/min in the treated cultures, divided by the mean ct/min in the untreated controls. For the mixed cell reactions, SI was defined as: (ct/min of $10^6$ blood lymphocytes mixed with $10^6$ mitomycin-treated tissue cells—ct/min of $10^6$ mitomycin-treated tissue cells alone) ÷ ct/min of $10^6$ untreated blood lymphocytes.

### RESULTS

### *Mixed-cell reactions*

In patients with malignant lymphoma in whom the spleen or lymph node was

TABLE I.—*Hodgkin's and Other Diseases: Tissue free of Tumour*

| Patient | Diagnosis | Age | Sex | Tissue source | Stimulation Index (SI) (mixed-cell cultures) | SI (homogenate-treated cultures) |
|---|---|---|---|---|---|---|
| (a) Hodgkin's Disease; tissue free of tumour | | | | | | |
| S.I. | Nodular sclerosing | 29 | F | Spleen | — | 1·95 |
| S.M. | Nodular sclerosing | 28 | F | Spleen | 0·3 | 1·4 |
| G.W. | Mixed cellularity | 57 | M | Spleen | — | 0·7 |
| J.M. | Mixed cellularity | 23 | M | Spleen | 0·4 | 0·9 |
| A.W. | Nodular sclerosing | 31 | F | Spleen | — | 1·5 |
| L.W. | Nodular sclerosing | 54 | M | Lymph node | 0·5 | — |
| H.M. | Mixed cellularity | 63 | M | Spleen | 0·5 | 1·2 |
| J.R. | Nodular sclerosing | 39 | F | Spleen | 1·4 | — |
| I.O. | Nodular sclerosing | 30 | F | Spleen | 0·6 | 1·0 |
| A.S. | Nodular sclerosing | 23 | M | Spleen | 1·6 | 0·6 |
| W.H. | Lymphocytic predominant | 57 | M | Spleen | 0·4 | 0·9 |
| D.I. | Mixed cellularity | 36 | M | Spleen | — | 1·5 |
| (b) Other non-neoplastic spleens | | | | | | |
| M.W. | Cholecystitis | 66 | F | Spleen | — | 0·8 |
| C.P. | Hypersplenism (reactive hyperplasia) | 64 | F | Spleen | 0·4 | 0·7 |

free of tumour, and in other non-neoplastic spleens (Table I), the stimulation index ranged from 0·4 to 1·6 with a mean of 0·7 (s.d. 0·47). From tables for " t " values it is calculated that 95% of results would be less than 1·5. A more conservative figure of 2·0 or above was taken to indicate a positive result, and all control values fell below this.

When lymphoma tissue was present in the spleen, 6 out of 14 patients (43%) showed a positive response (Table II, Fig.). The indices of 4·8, 20·6 and 6·2 are highly significant ($P < 0{\cdot}001$), the first 2 being obtained from nodular sclerosing Hodgkin's disease, and the third from follicular lymphoma of lymphocytic type. The 3 indices of 2·2 were

TABLE II.—*Malignant Lymphomas: Tumour Present in Tissue*

| Patient | Diagnosis | Age | Sex | Tissue source | SI (mixed-cell culture) | SI (homogenate-treated cultures) |
|---|---|---|---|---|---|---|
| (a) Hodgkin's disease | | | | | | |
| P.D. | Mixed cellularity | 36 | M | Spleen | — | 7·0 |
| D.L. | Nodular sclerosing | 33 | F | Spleen | — | 18·0 |
| G.B. | Mixed cellularity | 21 | M | Spleen | 0·6 | 1·4 |
| D.G. | Nodular sclerosing | 31 | M | Spleen | 4·8 | 1·6 |
| M.M. | Nodular sclerosing | 42 | F | Spleen | — | 1·8 |
| M.H. | Lymphocyte depleted | 21 | M | Spleen | 2·2 | 1·1 |
| A.M. | Nodular sclerosing | 40 | M | Spleen | 20·6 | 0·5 |
| A.K. | Mixed cellularity | 29 | M | Spleen | 0·9 | 1·4 |
| W.H. | Mixed cellularity | 45 | M | Spleen | 0·5 | 0·6 |
| W.C. | Nodular sclerosing | 63 | M | Spleen | 0·8 | 2·3 |
| S.H. | Nodular sclerosing | 23 | F | Spleen | 0·7 | 0·6 |
| (b) Other lymphomas | | | | | | |
| P.W. | Lymphocytic lymphoma (well differentiated) | 49 | F | Spleen | 2·1 | 2·3 |
| B.G. | Lymphocytic lymphoma (well differentiated) | 62 | F | Spleen | 0·7 | 0·6 |
| C.N. | Lymphocytic lymphoma (poorly differentiated) | 33 | F | Spleen | — | 2·3 |
| G.A. | " Histiocytic " lymphoma | 50 | F | Spleen | 0·6 | — |
| T.D. | Lymphocytic lymphoma (well differentiated) | 62 | M | Lymph node | — | 4·5 |
| B.C. | Lymphocytic lymphoma (follicular) | 50 | F | Spleen | 6·2 | — |
| J.M. | " Histiocytic " lymphoma | 60 | F | Spleen | 2·2 | — |
| D.T. | Lymphocytic lymphoma | 50 | M | Spleen | 0·4 | 0·4 |

FIG.—Response of autologous lymphocytes to stimulation (SI) by: A, whole cells from control spleens; B, whole cells from tumour deposits; C, homogenate of control spleens; D, homogenate of tumour deposits.

obtained from well-differentiated lymphocytic lymphoma, lymphocyte-depleted Hodgkin's disease and " histiocytic " lymphoma.

*Homogenate-treated cultures*

With tumour-free spleens from patients with malignant lymphoma and with other non-neoplastic spleens (Table I), the stimulation index ranged from 0·6 to 1·9 with a mean of 1·1 (s.d. 0·4). From " t " tables, 95% of these control values would be expected to be 1·8 or below, so an index of 2·0 or above was regarded as positive, as for the mixed-cell reactions, and all control figures fell in the negative range.

Positive reactions were seen in 7 out of 16 patients (44%) in whom tumour was present in the spleen (Table II, Fig.). The indices of 18·0, 7·0 and 4·5 are highly significant ($P < 0{\cdot}001$) and are from nodular sclerosing Hodgkin's disease, mixed cellularity Hodgkin's disease and lymphocytic lymphoma. The 2 figures of 2·3 are significant at the 1% level, and relate to nodular sclerosing Hodgkin's disease and lymphocytic lymphoma. The SI of 2·0 was from nodular sclerosing Hodgkin's disease, and is significant at the 5% level.

*Phytohaemagglutinin-treated cultures*

PHA-treated cultures invariably showed some degree of stimulation, with a maximum of 42,000 ct/min and an SI ranging from 2·0 to 180. A detailed protocol of one experiment is provided in Table III.

*Heterologous reactions*

Using a panel of 10 normal donors, positive reactions were obtained in a total of 34 out of 99 tests (34%) in which allo-

TABLE III.—*Patient A.M., Nodular Sclerosing Hodgkin's Disease with Splenic Deposits. Testing of Spleen Cell Suspension*

| | ct/min (mean of 3 cultures) |
|---|---|
| Blood lymphocytes alone | 170 (3 days) |
| | 80 (7 days) |
| Blood lymphocytes + PHA | 8200 (3 days) |
| Blood lymphocytes + mitomycin-treated spleen cells | 1700 (7 days) |
| Mitomycin-treated spleen cells alone | 50 (7 days) |

$$\text{SI (PHA)} = \frac{8200}{170} = 48{\cdot}2$$

$$\text{SI (spleen cells)} = \frac{1700 - 50}{80} = 20{\cdot}6$$

geneic lymphocytes were treated with a range of spleen homogenates. When tumour was present in the spleen extract, 21 out of 69 (30%) were positive (SI 2·1–44, mean 10·5). With tumour-free splenic extracts, 13 out of 30 tests (43%) were positive (SI 3·2–50, mean 17·2).

### DISCUSSION

The results demonstrate significant autologous lymphocyte blastogenesis, in response to both suspended lymphoma cells and tumour homogenates, compared with the effects of similar preparations of non-malignant spleens. The present investigation has concentrated exclusively on malignant lymphomas, but the results are comparable with those obtained from studies of other solid tumours. Lymphocyte stimulation was demonstrated in 10 out of 18 patients with various non-lymphomatous malignant tumours, in response to whole tumour cells, and 9 of the 10 patients with positive reactions also responded to *in vitro* stimulation with a potassium chloride extract of tumour tissue (Vanky *et al.*, 1974). In a similar analysis, 19 out of 29 patients with malignant tumours, which included a few lymphomas, showed positive reactions between tumour cells and patient's lymphocytes (Mavligit *et al.*, 1973) and 2 patients responded to a tumour extract. A smaller proportion of positive results was found by Savel (1969), who obtained significant stimulation in 7 patients out of 56, using a saline extract of tumour; 6 lymphomas of unspecified type were amongst the negative responders and no lymphomas were positive. Positive *in vitro* reactions have been found in lymphosarcoma and Hodgkin's disease, using the M.I.F. method (Braun *et al.*, 1972).

In experimental situations of the type we have investigated, a useful control would be to compare the effects of tumour cells and normal cells from the same organ. This is possible if there are reasonably large areas of tumour-free spleen, in addition to the presence of tumour nodules. Our experience to date has been that whenever large dissectable tumour nodules have been present, small nodules have usually been present in the intervening splenic tissue, with the result that it has been possible to perform this control on one occasion only. In this instance, both tumour-containing and tumour-free areas responded negatively.

In the present study, 11 out of 19 patients had a positive response in at least one of the tests employed. It is of interest that of these 11 positive results, 4 were from patients with nodular sclerosing Hodgkin's disease, and only 2 patients with nodular sclerosis had a negative result (Table II). The numbers are too small to draw any definite conclusions in relation to individual histological types, but it is of note that nodular sclerosis is one of the subvarieties of Hodgkin's disease carrying a better prognosis than average (Lukes, Butler and Hicks, 1966*a*; Gough, 1970).

Possible explanations for the reactions are: (a) that blastogenesis occurs in lymphocytes which were presensitized *in vivo* to an antigenic component of the tumour cell; (b) that lymphocytic stimulation represents a primary immune reaction to a tumour-associated antigen; (c) that lymphocytic stimulation is caused by a non-specific mitogen of no immunological significance.

The third explanation could not apply to the mixed-cell reaction, and appears very unlikely in the case of tissue extracts, in view of the fact that no significant stimulation occurs using extracts of normal autologous spleen.

In the mixed-cell reactions, it is not possible to be certain whether a primary or secondary immune reaction is at work, but the latter appears at least to contribute, in view of the finding that the blastogenic response to leukaemic cells can be increased by immunization with irradiated leukaemic cells during remission (Powles *et al.*, 1971).

It has been generally thought that subcellular preparations of disrupted cells stimulate allogeneic lymphocytes poorly or not at all (Hardy, Ling and Knight,

1969), and that lymphocytic blastogenesis to a solubilized extract represents a secondary immune response. The situation with tumour extracts is more complicated, in that while some investigators found that tumour extracts do not produce stimulation of normal lymphocytes (Jehn, 1970; Vanky *et al.*, 1974), Dean *et al.* (1975) have recently demonstrated that breast carcinoma extracts can stimulate lymphocytes from normal individuals. They regarded this as a primary immune response to normal alloantigens in the extract, and concluded that investigation of blastogenic responses to breast carcinoma should be restricted to autologous situations. Similar results are evident from the present study, in which blastogenesis can be induced in normal allogeneic lymphocytes by spleen extracts with or without tumour deposits. As no significant stimulation occurs in autologous lymphocytes in response to tumour-free splenic extracts, the interpretation that autologous blastogenesis produced by tumour-containing extracts represents an immunological event, remains valid, but it is not possible to distinguish between a primary and secondary immune response. The leucocyte migration inhibition factor assay is influenced by alloantigens to a lesser degree (McCoy *et al.*, 1974), and may prove to be a more suitable index of heterologous reactions.

The degree of lymphocytic stimulation by whole tumour cells and homogenates is less than that obtained by stimulation with allogeneic lymphocytes, with some individual exceptions (Powles *et al.*, 1971), indicating that, generally speaking, the antigenic difference between tumour cells and autologous somatic cells is less than the antigenic difference between cells of unrelated individuals. It appears, however, that at least in leukaemia, the difference is sufficient to make immunotherapy using killed autologous tumour cells a realistic proposition (Powles *et al.* 1973).

If immunotherapy using tumour cells or extracts should be considered for the therapy of malignant lymphomas, laboratory evidence of immunological differences between host and tumour cells is a useful prerequisite to providing a rational basis for treatment.

This work was supported by Clinical Research Grant CL177 provided by the South Glamorgan Area Health Authority.

We are grateful to Dr J. Whittaker and Mr D. Crosby for allowing us to investigate patients under their care.

### REFERENCES

Bach, F. H. & Voynow, N. K. (1966) One Way Stimulation in Mixed Leucocyte Cultures. *Science, N.Y.*, **153**, 545.

Braun, M., Sen, L., Bachmann, A. E. & Pavlovsky, A. (1972) Cell Migration Inhibition in Human Lymphomas using Lymph Node and Cell-line Antigens. *Blood*, **39**, 368.

Dean, J. H., Silva, J. S., McCoy, J. L., Leonard, C. M., Middleton, M., Cannon, G. B. & Herberman, R. B. (1975) Lymphocyte Blastogenesis Induced by Potassium Chloride Extracts of Allogeneic Breast Carcinoma and Lymphoid Cells. *J. natn. Cancer Inst.*, **54**, 1295.

Gough, J. (1970) Hodgkin's Disease; a Correlation of Histopathology with Survival. *Int. J. Cancer*, **5**, 273.

Hardy, D. A., Ling, N. R. & Knight, S. C. (1969) Exceptional Lymphocyte Stimulating Capacity of Cells from Lymphoid Cell Lines. *Nature, Lond.*, **223**, 511.

Hellstrom, I., Hellstrom, K. E., Sjogren, H. O. & Warner, G. A. (1971) Demonstration of Cell Mediated Immunity to Human Neoplasms of Various Histological Types. *Int. J. Cancer*, **7**, 1.

Jehn, V. W. (1970) *In vitro* Lymphocyte Stimulation by a Soluble Antigen from Malignant Melanoma. *New Engl. J. Med.*, **283**, 329.

Lewis, M. G. (1967) Possible Immunological Factors in Human Malignant Melanoma in Uganda. *Lancet*, ii, 921.

Lukes, R. J., Butler, J. J. & Hicks, E. B. (1966*a*) Natural History of Hodgkin's Disease as Related to its Pathological Picture. *Cancer, N.Y.*, **19**, 317.

Lukes, R. J., Craver, L., Hall, T., Rappaport, H. & Ruben, P. (1966*b*) Report of the Nomenclature Committee, Symposium on the Obstacles to the Control of Hodgkin's Disease. *Cancer Res.*, **26**, 1311.

McCoy, J. L., Jerome, L. F., Dean, J. H., Cannon, G. B., Alford, T. C., Doering, T. & Herberman, R. B. (1974) Inhibition of Leukocyte Migration by Tumour-associated Antigens in Soluble Extracts of Human Breast Carcinoma. *J. natn. Cancer Inst.*, **53**, 11.

Mavligit, G. M., Gutterman, J. U., McBride, C. M. & Hersh, E. M. (1973) Cell Mediated Immunity to Human Solid Tumours: *In vitro* Detection by Lymphocyte Blastogenic Responses to Cell Associated and Solubilized Tumour Antigens. *Natn Cancer Inst. Monogr.*, **37**, 167.

MORTON, D. L., MALMGREN, R. A., HOLMES, E. C. & KETCHAM, A. S. (1968) Demonstration of Antibodies against Human Malignant Melanoma by Immunofluorescence. *Surgery*, **64**, 233.

POWLES, R. L., BALCHIN, L. A. & FAIRLEY, G. H. (1971) Recognition of Leukaemic Cells as Foreign Before and After Auto-immunization. *Br. med. J.*, i, 486.

POWLES, R. L., CROWTHER, D., BATEMAN, C. J., BEARD, M. E., MCELWAIN, T. J., RUSSELL, J., LISTER, T. A., WHITEHOUSE, J. M., WRIGLEY, P. F., PIKE, M., ALEXANDER, P. & FAIRLEY, G. H. (1973) Immunotherapy for Acute Myelogenous Leukaemia. *Br. J. Cancer*, **28**, 365.

RAPPAPORT, H. (1966) *Tumours of the Haematopoietic System*. Armed Forces Institute of Pathology.

SAVEL, H. (1969) Effect of Autologous Tumour Extracts on Cultured Human Peripheral Blood Lymphocytes. *Cancer, N.Y.*, **24**, 56.

VANKY, F., KLEIN, E., STJERNSWARD, J. & NILSONNE, U. (1974) Cellular Immunity Against Tumour Associated Antigens in Humans: Lymphocyte Stimulation and Skin Reaction. *Int. J. Cancer*, **14**, 277.